# Dry nettle (*Urtica dioica*) as a feed additive: impacts on duck growth, meat quality, gut morphology, gene expression, and microbiota

**DOI:** 10.1186/s12917-026-05537-1

**Published:** 2026-05-07

**Authors:** Sebastian Wlaźlak, Konrad Edwartowski, Weronika Krajniak, Zofia Siuta, Natalia Pawlikowska, Nikola Piotrowska, Nikola Śmiłowicz, Konrad Pilarski, Jakub Bielec, Sebastian Knaga, Patrycja Reszka, Iwona Zaremba, Aleksandra Dunisławska, Mirosław Banaszak, Marek Adamski, Jakub Biesek

**Affiliations:** 1 Department of Animal Breeding and Nutrition, Faculty of Animal Breeding and Biology, Bydgoszcz University of Science and Technology, Mazowiecka 28, Bydgoszcz, 85-084 Poland; 2 AVIUM Student Scientific Club, Faculty of Animal Breeding and Biology, Bydgoszcz University of Science and Technology, Mazowiecka 28, Bydgoszcz, 85-084 Poland; 3 Department of Animal Biotechnology and Genetics, Faculty of Animal Breeding and Biology, Bydgoszcz University of Science and Technology, Mazowiecka 28, Bydgoszcz, 85-084 Poland

**Keywords:** Waterfowl, Gastrointestinal tract, Health status, Meat quality, Phytogenic additive

## Abstract

**Background:**

This study evaluated the effects of dried nettle (*Urtica dioica*) supplementation on broiler ducks. A total of 180 Cherry Valley ducks were assigned to three groups: control, 1% nettle, and 3% nettle. Growth performance, carcass traits, meat quality, intestinal morphology, digesta viscosity, liver gene expression, and cecal microbiota were assessed. During rearing, the ducks’ body weight, growth, feed intake, and conversion ratio were controlled and calculated. After slaughter, carcass composition, meat quality, duodenum and jejunum morphology, liver gene expression, and intestinal microbiota composition were analyzed.

**Results:**

Nettle supplementation did not influence body weight, feed intake, feed conversion ratio, European Broiler Index, carcass yield, organ weights, or meat quality parameters. However, notable improvements in intestinal structure were observed, especially in ducks receiving 3% nettle, which showed increased duodenal villus width and surface area, as well as higher jejunal villus height. Digesta viscosity was significantly reduced in the jejunum of the 3% nettle group. Gene expression analysis revealed significant upregulation of *CYP7A1* in the 1% nettle group and ALB in the 3% group. Antioxidant-related genes (*SOD1* and *SOD2*) were significantly upregulated, and *GPX2* downregulated, only in the 3% nettle group, with no significant changes observed in the 1% group. Although beneficial bacterial populations were unchanged, a marked reduction in *Fusobacterium nucleatum* was detected in the 3% group, indicating a selective antimicrobial effect.

**Conclusions:**

Dried nettle at 3% improved gut morphology and antioxidant responses without affecting growth or meat quality, supporting its potential as a functional feed additive.

## Background

Broiler duck production plays a substantial role in the global poultry industry, with Asian countries accounting for over 80% of total output [1], and is still growing in the EU, with leaders: France, Hungary, and Poland [2]. Duck meat is distinguished by its unique nutritional profile, characterized by higher intramuscular fat content and a distinct flavor compared to chicken. At the same time, increasing consumer awareness of food safety and quality has intensified the search for alternatives to antibiotic growth promoters (AGP), whose prolonged use has contributed to antimicrobial resistance and raised public health concerns [3, 4]. The EU and other regions have consequently imposed restrictions or bans on AGP in animal nutrition, accelerating the development of natural feed additives [5].

Among phytogenic feed additives, medicinal herbs have attracted growing interest due to their bioactive compounds that may improve productivity and health without adverse side effects [6]. *Urtica dioica* L. (common nettle) is one such herb, rich in vitamins (A, C, K), minerals (iron, calcium, magnesium), polyphenols, and chlorophyll, which collectively exert antioxidant, anti-inflammatory, and immunomodulatory effects [7]. Nettle has been traditionally used in animal feeding to support digestion, enhance feed intake, and improve immune responses [8]. Studies in broilers have demonstrated that dietary supplementation with dried nettle leaf powder can improve growth performance, feed efficiency, carcass traits, and selected biochemical parameters [9–12].

Hamedi et al. [13] confirmed the positive effects of a 1% nettle supplement on the histological structure of immune tissues in the cecal tonsils of broiler chickens. The observed changes included increased nodular unit width, a higher quantity of follicles per nodular unit, and greater muscular layer thickness. According to Sadeeq et al. [14], nettle can serve as a feed additive that supports growth and mitigates the negative effects of heat stress in Ross 308 broiler chickens. Birds fed diets supplemented with 1.5% and 3% nettle showed increased villus height compared to the control group. Moreover, in the 3% group, the villus height-to-crypt depth ratio was also significantly higher. Additionally, antibodies against Newcastle disease virus increased with higher nettle supplementation, rising from 5,666 in the control group to 9,701 in the group receiving 3% nettle. The other study has also evaluated the use of nettle extracts in drinking water. Behboodi et al. [15] found that administering 0.25 ml of nettle extract per 1 L of water to Ross 308 chickens was the optimal dose, positively influencing blood serum biochemistry and oxidative status. Despite the growing interest in phytogenic supplements, most studies have focused on broiler chickens, while data on ducks remain limited. Furthermore, few studies combine the simultaneous assessment of morphological, molecular, and microbiological parameters of the gastrointestinal tract.

The research hypothesis is that the addition of 1% and 3% dried nettle to the commercial diet affects the production performance, intestinal morphology, health status, and meat quality of broiler ducks. The study aimed to evaluate the impact of adding 1% and 3% dried nettle to the commercial diet on production performance, intestinal morphology, gene expression, cecal microbiota, and physicochemical quality characteristics of carcasses and meat of broiler ducks.

## Methods

The research was approved by the Committee for the Care of Animals of the Bydgoszcz University of Science and Technology, Poland (approval no. 1/24) and conducted according to the relevant regulations. The methods aligned with the ARRIVE principles [16]. The ducks used in the experiment were purchased from a commercial waterfowl hatchery and subsequently transported to the experimental farm where the study was conducted. As the animals were not privately owned by another institution or individual, no additional owner consent was required.

### Experimental design and animal rearing

The experiment was conducted on 180 one-day-old Cherry Valley commercial hybrid broiler ducks with an initial average body weight of 50.09 g. The birds were randomly assigned to three dietary treatment groups, each comprising six replicates with 10 birds per replicate. The experimental design included: a control group (**C**), receiving a commercial basal diet without additives; an experimental group (**N1**), receiving the same diet supplemented with 1% dried nettle; and a second experimental group (**N3**), receiving the basal diet supplemented with 3% dried nettle. Dried nettle did not replace any of the ingredients of the basal diet. The dry nettle was commercially available and mixed with granules.

The ducks were reared in floor pens, each covering an area, resulting in a maximum stocking density of 17 kg/m². The pens were constructed using tin-coated metal mesh. Environmental parameters were maintained following standard rearing guidelines: the ambient temperature was initially set at an average of 26 °C, with additional heating up to 30 °C during the early phase and a gradual reduction to 20 °C by the fourth week. Relative humidity was maintained at approximately 65%. Chopped wheat straw was used as a bedding material. Detailed environmental management has been described previously [17].

Birds had ad libitum access to fresh water and were fed according to the assigned dietary treatments. Water was supplied via two nipple drinkers per 10 birds. The feeding regimen was divided into two phases: a starter phase (days 1–28), during which a commercial starter diet was provided, followed by a grower phase (days 29–42) with a commercial grower diet. Both diets were offered in granulated form and formulated to meet the nutritional requirements of broiler ducks, being isocaloric and isonitrogenous, as per manufacturer specifications (De Heus, Łęczyca, Poland).

### Analytical composition of the commercial diet and dry nettle

The chemical composition of the commercial starter and grower diets, dry nettle and experimental diets (starter and grower) is presented in Table 1. Each material was analyzed in six replicates to determine its analytical profile. Dry matter (DM) was determined using a laboratory dryer (POL-EKO, Wodzisław Śląski, Poland) [18]. Crude ash (CA) content was measured gravimetrically based on the same standard. Crude fat (ether extract, EE) was analyzed using the Soxhlet method [19] (SOXTEC SYSTEM HIT 1043, Gemini BV, Apeldoorn, Netherlands). Crude protein (CP) was determined by the Kjeldahl method [20] (Kjeltec™ 8400 Analyzer Unit and Kjeltec Sampler 8420, FOSS, Hilleroed, Denmark). Acid detergent fiber (ADF, including residual ash) was analyzed [21]. Neutral detergent fiber (NDF, with heat-stable amylase and residual ash) was assessed [22] using the ANKOM 220 apparatus (Ankom, Macedon, NY, USA). Gross energy (GE) content was measured with an isoperibolic bomb calorimeter (KL-21 PLUS; Precyzja-Bit PPHU Ltd., Bydgoszcz, Poland) [23]. All analytical results are expressed per 1 kg of dry matter (DM) basis [24].

**Table 1:** Basal analytical composition of the commercial diet and dry nettle for broiler ducks

Ingredient	Commercial diet						
	Starter	Grower	Dry nettle	Starter + 1% dry nettle	Grower + 1%dry nettle	Starter + 3% dry nettle	Grower + 3% dry nettle
g/kg feed							
DM	908.32	883.47	899.63	908.23	883.63	908.06	883.96
g/kg DM							
Crude ash	57.36	45.04	118.93	57.98	45.78	59.21	47.26
Crude protein	215.94	199.29	161.45	215.40	198.91	214.31	198.16
Crude fat	37.16	37.84	14.57	36.93	37.61	36.48	37.14
Crude fiber	54.29	51.58	379.61	57.54	54.86	64.05	61.42
ADF	65.97	71.10	443.90	69.75	74.83	77.31	82.28
NDF	283.01	258.09	584.57	286.03	261.36	292.06	267.88
ADL	17.48	24.98	98.79	18.29	25.72	19.92	27.19
MJ/kg DM							
Gross energy	18.49	18.81	17.24	18.48	18.79	18.45	18.76

According to the manufacturer’s declaration, the starter feed contains the following ingredients: maize, wheat, soybean extraction meal, wheat bran, sunflower extraction meal, hulled sunflower seeds, barley, rapeseed extraction meal, wheat gluten feed, calcium carbonate, animal fat, monocalcium phosphate, vegetable oil (raw sunflower), sodium chloride, and sodium sulfate. The grower feed includes maize, wheat, wheat bran, soybean extraction meal, sunflower extraction meal (from dehulled sunflower seeds), triticale, rapeseed extraction meal, animal fat, calcium carbonate, monocalcium phosphate, sodium chloride, and calcium bicarbonate. The nutrient concentrations for the starter and grower feeds are as follows: Lysine: 9.30 g/kg (starter) and 8.70 g/kg (grower); Methionine: 4.20 g/kg (starter) and 3.70 g/kg (grower); Threonine: 7.20 g/kg (starter) and 6.10 g/kg (grower); Calcium: 8.50 g/kg (starter) and 8.10 g/kg (grower); Total phosphorus: 6.90 g/kg (starter) and 6.60 g/kg (grower); Sodium: 1.70 g/kg (starter) and 1.60 g/kg (grower); Vitamin A: 10,000 IU (both); Vitamin D_3_: 3,000 IU (both); Vitamin E: 25 IU

^1^, *DM* Dry matter, *ADF* Acid detergent fiber, *NDF* Neutral detergent fiber, *ADL* Acid detergent lignin

### Growth performance

Mortality was monitored daily throughout the rearing period, and viability was calculated as the percentage of surviving birds. Body weight (BW) was recorded on days 1, 28, and 42. Based on these measurements, growth rate (GR) and body weight gain (BWG) were determined. Growth rate was calculated using the formula: ($\:\frac{\mathrm{f}\mathrm{i}\mathrm{n}\mathrm{a}\mathrm{l}\:\mathrm{b}\mathrm{o}\mathrm{d}\mathrm{y}\:\mathrm{w}\mathrm{e}\mathrm{i}\mathrm{g}\mathrm{h}\mathrm{t}\:\left(\mathrm{g}\right)\:-\:\mathrm{i}\mathrm{n}\mathrm{i}\mathrm{t}\mathrm{i}\mathrm{a}\mathrm{l}\:\mathrm{b}\mathrm{o}\mathrm{d}\mathrm{y}\:\mathrm{w}\mathrm{e}\mathrm{i}\mathrm{g}\mathrm{h}\mathrm{t}\:\left(\mathrm{g}\right)}{0.5\:\times\:\:(\mathrm{i}\mathrm{n}\mathrm{i}\mathrm{t}\mathrm{i}\mathrm{a}\mathrm{l}\:\mathrm{b}\mathrm{o}\mathrm{d}\mathrm{y}\:\mathrm{w}\mathrm{e}\mathrm{i}\mathrm{g}\mathrm{h}\mathrm{t}\:\left(\mathrm{g}\right)\:+\:\mathrm{f}\mathrm{i}\mathrm{n}\mathrm{a}\mathrm{l}\:\mathrm{b}\mathrm{o}\mathrm{d}\mathrm{y}\:\mathrm{w}\mathrm{e}\mathrm{i}\mathrm{g}\mathrm{h}\mathrm{t}\:\left(\mathrm{g}\right)}\times\:100$) and body weight gain (BWG) ($\:\mathrm{f}\mathrm{i}\mathrm{n}\mathrm{a}\mathrm{l}\:\mathrm{b}\mathrm{o}\mathrm{d}\mathrm{y}\:\mathrm{w}\mathrm{e}\mathrm{i}\mathrm{g}\mathrm{h}\mathrm{t}\:\left(\mathrm{g}\right)-\mathrm{i}\mathrm{n}\mathrm{i}\mathrm{t}\mathrm{i}\mathrm{a}\mathrm{l}\:\mathrm{b}\mathrm{o}\mathrm{d}\mathrm{y}\:\mathrm{w}\mathrm{e}\mathrm{i}\mathrm{g}\mathrm{h}\mathrm{t}\:\left(g\right)$). Daily feed intake (FI) was recorded, and the feed conversion ratio (FCR) was calculated as: $\:\frac{\mathrm{f}\mathrm{e}\mathrm{e}\mathrm{d}\:\mathrm{i}\mathrm{n}\mathrm{t}\mathrm{a}\mathrm{k}\mathrm{e}\:\left(\mathrm{k}\mathrm{g}\right)}{\mathrm{b}\mathrm{o}\mathrm{d}\mathrm{y}\:\mathrm{w}\mathrm{e}\mathrm{i}\mathrm{g}\mathrm{h}\mathrm{t}\:\mathrm{g}\mathrm{a}\mathrm{i}\mathrm{n}\:\left(\mathrm{k}\mathrm{g}\right)}$. To assess overall production efficiency, the European Broiler Index **(**EBI**)** was calculated using the following formula: $\frac{\mathrm{viability}\left(\%\right)\;\times\;\mathrm{average}\;\mathrm{daily}\;\mathrm{gain}\left(\mathrm{kg}\right)}{\mathrm{feed}\;\mathrm{coversation}\;\mathrm{ratio}\left(\frac{\mathrm{kg}}{\mathrm{kg}}\right)}{\times100}$.

### Carcass characteristics and meat quality

After the 42-day rearing period, 12 ducks from each experimental group were selected for slaughter. Birds were chosen based on body weight, with individuals representing the average weight within each pen (two birds per pen), to ensure uniformity of the slaughter sample. Slaughter was carried out in compliance with Council Regulation (EC) No. 1099/2009 on the protection of animals at the time of killing. Birds were stunned by the electrical method, authorized for use in poultry, was applied. Following stunning, the birds were killed in compliance with the same regulatory requirements, followed by cervical dislocation at the junction between the first cervical vertebra and the occipital condyle.

Carcasses were defeathered using an automated plucking system and eviscerated. The feet were removed at the level of the hock joint. Carcasses were subsequently chilled at 4 ± 1 °C for 24 h in a refrigerator (Hendi, Poznań, Poland). After chilling, each carcass and edible offal (heart, liver, and gizzard) were weighed using a calibrated weight (Radwag, Radom, Poland). Simultaneously, the pH of the pectoralis major muscle was measured using a pH meter equipped with a spear-type electrode.

Carcass dissection was performed according to the methodology described by Ziołecki and Doruchowski [25]. The following carcass components were separated: neck (skinless), pectoral muscles (m. pectoralis major and minor), leg muscles (boneless thigh and drumstick), skin with subcutaneous fat (including neck skin), abdominal fat, wings with skin, and residual carcass components (trunk and leg bones). All individual components were weighed using the same weight. Slaughter performance was evaluated by calculating carcass yield, both with and without edible offal. Samples were calculated as g/kg of carcass or live body weight.

Color parameters of the pectoral and leg muscles were assessed using the CIELab color space system. Measurements of lightness (L*), redness (a*), and yellowness (b*) were obtained with a calibrated colorimeter (Konica Minolta, Tokyo, Japan) on the inner surface of both muscle groups. Drip loss was determined for the right pectoral muscle. Each sample was initially weighed, then placed in a double plastic bag and suspended in a refrigerated chamber at 4 ± 1 °C for 24 h. After this period, the muscle was reweighed, and drip loss was calculated as the percentage of weight loss relative to the initial muscle weight: $\:(100-(\frac{final\:sample\:weight\:\left(g\right)}{initial\:sample\:weight\:\left(g\right)})\times\:100)$. Water-holding capacity (WHC) was determined using a method analogous to that used for drip loss. Left pectoral and leg muscles were homogenized using a laboratory meat grinder (Hendi, Poznań, Poland). Subsequently, 0.295–0.305 g of ground muscle tissue was placed between two sheets of Whatman No. 1 filter paper and compressed under a constant load of 2 kg for 5 min. All meat quality assessments were conducted in 12 replicates per treatment group.

### Histological analysis of the small intestine

Samples (approx. 2 cm) for histological analysis were collected immediately after slaughter from two sections of the small intestine – the middle part of the duodenum and the jejunum. The tissue samples were fixed in Bouin’s solution, then dehydrated in alcohol, cleared in xylene, and embedded in paraffin using a tissue processor (Thermo Shandon, Chadwick Road, Astmoor, Runcorn, Cheshire, United Kingdom). Using an embedding station (Medite, Burgdorf, Germany), the samples were embedded into paraffin blocks and sectioned with a rotary microtome (Thermo Shandon, Chadwick Road, Astmoor, Runcorn, Cheshire, United Kingdom) into 7 μm-thick slices. PAS staining [26] was performed to assess the villus height and width, villus surface area, and crypt depth.

A Delta Optical Evolution 300 microscope equipped with a ToupCam™ camera and ToupView software was used for capturing microscopic images of the small intestine. Measurements of villus height, villus width, and crypt depth were performed using the MultiScan v. 18.03 software (Computer Scanning Systems II Sp. z o.o., Warsaw, Poland). The height and width of intestinal villi were measured on ten randomly selected villi. Height was measured from the top to the base of the villus, at the opening of the intestinal crypts. The width of the villi was measured at the midpoint of their height. The depth of the intestinal crypts was measured between 10 villi. The surface area of the villi was calculated based on the formula provided by Sakamoto et al. [27]: (2π) × (VW / 2) × (VH), where VW = villus width, and VH = villus height.

### Viscosity of the digesta of broiler ducks

Viscosity analyses of intestinal contents were performed using a modified protocol based on the method described by Pestana et al. [28]. Approximately 10 mL of jejunal and ileal digesta were collected from 10 birds into 15 mL Falcon tubes. Meckel’s diverticulum was used as the anatomical reference point to distinguish between the jejunum and the ileum. The samples were centrifuged using a laboratory centrifuge (Eppendorf, Hamburg, Germany) at 4,200 rpm for 10 min. A 1.5 mL aliquot of the resulting supernatant was collected using an automatic pipette (Chemland, Cracow, Poland) and transferred to 1.5 mL Eppendorf tubes. Viscosity measurements were conducted using a Brookfield cone-plate viscometer, model DVNext (LaboPlus, Warsaw, Poland). A 0.5 mL portion of the supernatant was applied to the measurement plate, and the analysis was performed at a rotation speed of 6 rpm. The final viscosity value for each sample was calculated as the mean of all readings obtained after 30 s of measurement. Each sample was analyzed in duplicate, resulting in 20 measurements per group.

### RNA and DNA Isolation

Total RNA was isolated from liver fragments collected from Cherry Valley broiler ducks and fixed in FixRNA (EURx, Gdansk, Poland). Approximately 100 mg of tissue was homogenized in 1 ml of RNA Extracol reagent (EURx, Gdansk, Poland) using a TissueRuptor homogenizer (Qiagen GmbH, Hilden, Germany). RNA was purified using the Universal RNA Purification Kit (EURx, Gdańsk, Poland), according to the manufacturer’s protocol. The quality and quantity of all RNA samples were assessed with a NanoDrop 2000 spectrophotometer (Thermo Scientific Nanodrop Products, Wilmington, USA). RNA isolates were stored at − 20 °C.

Total bacterial DNA was extracted from cecal digesta collected from the ducks on the day of slaughter. The material was placed into 15 ml tubes, immediately frozen in dry ice, and subsequently stored at − 80 °C. Approximately 100 mg of digesta was subjected to lysis and purified using the Stool DNA Purification Kit (EURx, Gdańsk, Poland), following the manufacturer’s instructions. The obtained DNA was evaluated spectrophotometrically using a NanoDrop 2000. DNA samples were then diluted to a working concentration of 20 ng/µl and stored at 4 °C until further analysis.

### Gene expression in liver tissue

Gene expression analysis was performed in the liver of ducks belonging to the control group (C) and two experimental groups receiving feed supplemented with 1% (N1) or 3% (N3) nettle (*Urtica dioica*). The analysis was conducted on samples collected from 8 individuals per group. Genes related to oxidative stress comprised *SOD1*, *SOD2*, and *GPX2*. Genes associated with lipid and cholesterol metabolism included *CYP7A1*, *HMGCR*, *ABHD5*, and *ACSL1*. The inflammatory response was represented by *IL-1β* and *CEBPB*, while *FST* was analyzed as a marker of cell growth and differentiation. Finally, genes related to energy metabolism and glycogen regulation included *PDK4*, *PHKB*, and *ALB*. The selection of genes for hepatic expression profiling was based on their key roles in major physiological pathways likely to be modulated by dietary bioactive compounds of plant origin, such as nettle. The analysis included genes representing four major functional categories: oxidative stress, lipid and cholesterol metabolism, inflammatory response and cellular growth, and energy and carbohydrate metabolism.

Normalization of target gene expression levels was performed relative to the geometric mean of two reference genes: *ACTB* (β-actin) and *GAPDH* (glyceraldehyde-3-phosphate dehydrogenase). Gene expression was assessed using two-step quantitative reverse transcription PCR (RT-qPCR).

### Gene expression analysis

For gene expression analysis, 3500 ng of total RNA from each sample was reverse-transcribed into cDNA using the smART First Strand cDNA Synthesis Kit (EURx, Gdańsk, Poland), following the manufacturer’s protocol. qPCR reactions were prepared in a total volume of 10.5 µl in a 384-well plate format. Each reaction contained SG onTaq qPCR Master Mix (EURx, Gdańsk, Poland), 0.2 µM of each primer (forward and reverse), and 140 ng of cDNA, with nuclease-free water added to reach the final volume. Oligonucleotide primers (Table 2) were designed using the NCBI Primer-BLAST tool. Where possible, amplicon sequences spanned exon-exon junctions.

**Table 2:** qPCR primer sequences used for the relative expression analysis of selected genes and universal and genus-/species-specific primer sequences

qPCR primer sequences used for the relative expression analysis of selected genes		
Gene symbol	Forward	Reverse
*SOD1*	TGGGGACAACACAAATGGATG	TCCAGTCAGAGAGATGACGG
*SOD2*	TGCAAGGAACAACAGGTCTT	TGGTGCCATTTCAGTAGTCTG
*GPX2*	TTTGGCTACCAGGAGAACGG	GCTTTCAGGTAGGCGAAGAC
*CYP7A1*	AAAAGGAGAGCCACCACTTG	CTTTGAGGAATTCGAGGGGG
*HMGCR*	CAGCACTAGTAGGTTTGCCC	TTGCATCCCCTGTTCTTGAC
*ABHD5*	GTAGCAGCGATGTAGGACAC	TGCAGAATGGGATCGTCTTG
*ACSL1*	GCTGTTGTGGTACCTGATCC	AGCTTGAAAGAGTTGATGCAGA
*IL-1β*	GCGAAGAGACCTTCTACGGC	TGGTGTGCTCAGAATCCAGG
*CEBPB*	ATGCTAAGGGGTCCAGGTAG	TATTACGAGGCGGACTGTCT
*FST*	AACTTACCCAAGCGAGTGTG	ACCTAGGGATTGCCTTCAGA
*PDK4*	CCACTAATGCAGTGAGGTGA	ACCAGTTAGAAAGTTTACTGGCA
*PHKB*	CTGTAGCTTAGCCTGAGACG	CAAAAGCACAAAACTGCACAC
*ALB*	GACTTCCTCAAGGCATTGCT	CTCAGAACAAGGTAGGCGTC
*ACTB*	CCGTAAGGACCTGTACGCCAACAC	GCTGATCCACATCTGCTGGAAGG
*GAPDH*	GGTTGTCTCCTGCGACTTCA	TCCTTGGATGCCATGTGGAC

Universal and genus-/species-specific primer sequences		
Genus/Species	Forward	Reverse
Universal	TCCTACGGGAGGCAGCAGT	GGACTACCAGGGTATCTAATCCGTT
*Lactobacillus spp.*	AGCAGTAGGGAATCTTCCA	CACCGCTACACATGGAG
*Salmonella spp.*	ATTCTGGTACTAATGGTGATGATC	GCCAGGCTATCGCCAATAAC
*Bifidobacterium spp.*	GCGTGCTTAACACATGCAAGTC	CACCCGTTTCCAGGAGCTATT
*Campylobacter spp.*	CTGAATTTGATACCTTAAGTGCAGC	AGGCACGCCTAAACCTATAGCT
*Streptococcus spp.*	AGATGGACCTGCGTTGT	GCTGCCTCCCGTAGGAGTCT
*Eschericha coli*	CATGCCCGCGTGTATGAAGAA	CGGGTAACGTCAATGAGCAAA
*Faecalibacterium rausnitzii*	ACCATGAGAGCCGGGGGG	GGTTACCTTGTTACGACTT
*Fusobacterium nucleatum*	AAGCGCGTCTAGGTGGTTATGT	TGTAGTTCCGCTTACCTCTCCAG

RT-qPCR reactions were performed using the LightCycler 480 system (Roche Diagnostics, Basel, Switzerland) under the following thermal cycling conditions: initial denaturation at 95 °C for 15 min, followed by 40 cycles of amplification (denaturation at 95 °C for 15 s, primer annealing at 58 °C for 20 s, and extension at 72 °C for 20 s), and a subsequent melting curve analysis. The annealing temperature was set to 58 °C for all genes, except for *IL12p40*, which required 65 °C. Fluorescence was measured at the end of each extension step. The melting curve was generated by gradually increasing the temperature to 98 °C while continuously monitoring fluorescence associated with amplicon dissociation. Relative gene expression was calculated using the ΔΔCt method, and the amount of target gene transcript was determined using the formula 2^–ΔΔCt [29].

### Quantification of bacteria by qPCR

The relative abundance of bacterial genera *Bifidobacterium*, *Lactobacillus*, *Streptococcus*, and *Salmonella*, as well as the species *Escherichia coli*, *Faecalibacterium prausnitzii*, and *Fusobacterium nucleatum*, in the cecal content of Cherry Valley commercial ducks was determined using quantitative PCR (qPCR), conducted with the LightCycler 480 II system (Roche Diagnostics, Basel, Switzerland). Targeted analysis of selected bacterial taxa was performed on cecal samples collected from 8 individuals per group. Each qPCR reaction had a total volume of 10.5 µl in a 96-well plate format and contained: 6.25 µl of SG onTaq qPCR Master Mix (EURx, Gdańsk, Poland), 0.2 µM of each primer specific to the 16 S rRNA gene of the targeted bacterial genus or species (Table 2), including universal bacterial primers, and 2 ng of bacterial DNA as template. All reactions were performed in two technical replicates.

The thermocycling protocol consisted of an initial denaturation at 95 °C for 5 min, followed by 40 amplification cycles: denaturation at 95 °C for 15 s, primer annealing at 58 °C for 30 s, and extension at 72 °C for 30 s. Fluorescence was measured at the end of each extension step. After amplification, a melting curve analysis was performed by gradually increasing the temperature to 98 °C while continuously measuring the fluorescence of the melting PCR products.

Mean Ct values from the two technical replicates, obtained using the LightCycler 480 II software, were used for data analysis. Ct values deviating by more than 0.3 from the replicate average were considered outliers. PCR efficiency for each primer pair was calculated using the LightCycler 480 II software based on a five-point dilution series (1x, 0.5x, 0.25x, 0.125x, and 0.0625x) of pooled bacterial DNA. The relative abundance of bacteria in the cecal content was calculated using the following formula:

Relative abundance [%] = (E_universal)^Ct_universal / (E_target)^Ct_target.

Where: E_universal – PCR efficiency using universal primers for total bacteria; Ct_universal – Ct value from the reaction with universal bacterial primers; E_target – PCR efficiency using primers specific to the target genus or species; Ct_target – Ct value from the reaction with target-specific primers.

### Statistical analysis

Statistical analyses were conducted using Statistica software version 13.3.0 (TIBCO Software Inc., Cracow, Poland). Mean values and pooled standard errors of the mean (SEM) were calculated. Variance homogeneity was verified using Levene’s test, confirming equal variances across groups. The Shapiro–Wilk test was applied to assess normality, indicating near-normal distribution of the dependent variables. For performance-related variables, the experimental unit used in the statistical analysis was the pen. One-way analysis of variance was used to evaluate treatment effects, based on the model: Yₓ = µ + Cₓ + eₓ, where *Yₓ* is the dependent variable, *µ* is the overall mean, *Cₓ* is the fixed effect of diet (commercial diet; commercial diet + 1% dried nettle; commercial diet + 3% dried nettle), and *eₓ* is the residual error. The interaction between nettle and intestine (viscosity of digesta) was also analyzed by two-way analysis of variance with statistical model: Y_NI_ = µ + C_N_ + D_I_ + CD_NI_ + e_NI_, where Y_N/I_, the dependent variable; µ, the overall mean; C_N_, the effect of nettle supplementation (C, N1, N3); D_I_, the impact of intestine (jejunum, ileum); CD_NI_, the interaction between N and I; e_NI_, residual error). Tukey’s post hoc test determined significant differences between means, with statistical significance set at *P* < 0.05 for growth, carcass, meat, and viscosity features. For histological parameters, Duncan’s post hoc test to identify significant differences was used with *P* < 0.05.

RT-qPCR data analysis was performed using SAS 9.4. mean Ct values were calculated, followed by ΔCt determination relative to the average Ct of the reference genes (*ACTB* and *GAPDH*). Gene expression levels were then assessed using the ΔΔCt method relative to the control group (K). The results were expressed as Fold Change (2^–ΔΔCt) and log₂ Fold Change (log₂FC). Normality of data distribution was assessed using the Shapiro–Wilk test, and differences between groups were analyzed using one-way ANOVA followed by Tukey’s post hoc test (*P* ≤ 0.05). The results were visualized as log₂FC plots.

Statistical analysis of microbial community composition was performed in R (version 2025.05.0) using the dplyr, ggpubr, and rstatix packages. Normality was assessed within each experimental group using the Shapiro–Wilk test. As the data did not meet normality assumptions (*P* < 0.05), differences between groups were evaluated using the non-parametric Kruskal–Wallis test. Pairwise comparisons were performed using Dunn’s post hoc test with Bonferroni correction (*P* ≤ 0.05).

## Results

### Growth performance

Dietary supplementation with dried nettle at 1% and 3% inclusion levels did not result in statistically significant differences in production performance parameters (*P* > 0.05). All assessed variables, including viability, body weight on days 1, 28, and 42, body weight gain, growth rate, feed intake, feed conversion ratio, and the European Broiler Index, remained comparable across groups. Final body weight on day 42 ranged from 3253.95 g in the N3 group to 3330.60 g in the control group (*P* = 0.582), and FCR ranged from 2.56 to 2.72 kg/kg (Table 3).

**Table 3:** Production results of broiler ducks

Item		Group			SEM	*P*-value^*^
		C	N1	N3		
Viability (%)		93.33	88.33	98.33	2.287	0.211
Body weight (g)	d 1	50.02	50.93	49.33	0.346	0.170
	d 28	1920.65	1952.47	1912.84	25.988	0.823
	d 42	3330.60	3322.62	3253.95	31.621	0.582
Growth rate (%)	d 1–28	189.82	189.76	189.94	0.167	0.916
	d 29–42	53.69	52.12	51.91	0.930	0.720
	d 1–42	194.07	193.95	194.02	0.069	0.779
Body weight gain (g)	d 1–28	1870.63	1901.53	1863.51	26.042	0.836
	d 29–42	1409.95	1370.15	1341.11	23.261	0.507
	d 1–42	3280.58	3271.69	3204.62	31.595	0.591
Feed intake (g)	d 1–28	3816.88	3991.38	3814.90	56.142	0.359
	d 29–42	4357.91	4485.69	4340.86	39.125	0.269
	d 1–42	8399.61	8902.27	8214.77	125.216	0.058
FCR (kg/kg)	d 1–28	2.05	2.12	2.05	0.044	0.793
	d 29–42	3.12	3.28	3.24	0.059	0.533
	d 1–42	2.56	2.72	2.56	0.035	0.089
European Broiler Index		285.72	254.61	292.60	9.247	0.210

*SEM* Standard error of the mean, *FCR* Feed conversation ratio, *C* Control group fed with a commercial diet, *N1* group fed with a commercial diet + 1% of dry nettle, *N3* group fed with a commercial diet + 3% of dry nettle

*no statistically significant differences, *P* value > 0.05

### Carcass characteristics and meat quality

No statistically significant differences between groups were observed in slaughter yield or carcass component proportions (*P* > 0.05). Slaughter yield ranged from 70.71% in the control group to 71.45% in the N3 group (*P* = 0.588). Similarly, the proportion of breast muscles varied from 18.25% to 19.16% (*P* = 0.501), and leg muscles ranged from 13.72% to 14.62% (*P* = 0.124). Other carcass elements and organ weights showed no significant variation attributable to dietary nettle supplementation. Physicochemical characteristics of breast and leg muscles, including pH measured 24 h postmortem, color parameters, drip loss, and WHC, were not significantly affected by the addition of dried nettle (*P* > 0.05) (Table 4).

**Table 4:** Carcass characteristics and meat quality of broiler ducks

Item	Group			SEM	*P*-value*
	C	N1	N3		
g/100 g of pre-slaughter body weight					
Slaughter yield	70.71	70.91	71.45	0.297	0.588
Slaughter yield with offals	77.22	77.52	78.16	0.338	0.528
g/100 g of carcass with offal					
Heart	0.76	0.73	0.73	0.013	0.726
Liver	3.82	3.98	3.94	0.094	0.774
Gizzard	3.85	3.80	3.90	0.084	0.877
g/100 g of carcass					
Neck	7.28	6.70	7.18	0.186	0.413
Breast muscle (BM)	18.25	19.16	18.46	0.324	0.501
Leg muscle (LM)	14.62	13.72	14.44	0.190	0.124
Skin with subcutaneous fat	20.76	21.26	21.63	0.373	0.646
Abdominal fat	0.67	0.92	0.89	0.057	0.151
Wings (with skin)	12.60	12.59	11.63	0.251	0.194
Carcass remains	25.81	25.64	25.77	0.457	0.988
Physicochemical features of breast and leg muscles					
pH_24_	5.88	5.89	5.92	0.016	0.553
Color of BM					
L*	40.38	40.30	40.40	0.504	0.997
a*	15.90	16.52	16.60	0.256	0.483
b*	2.92	3.26	3.55	0.154	0.251
Color of LM					
L*	40.88	39.11	39.53	0.429	0.216
a*	15.43	15.35	14.59	0.287	0.437
b*	4.00	3.74	3.61	0.156	0.605
%					
Drip loss of BM	1.20	1.31	1.15	0.154	0.916
WHC of BM	37.16	37.98	35.33	0.775	0.369
WHC of LM	33.91	32.96	31.74	0.491	0.200

*SEM* Standard error of the mean, *C* Control group fed with a commercial diet, *N1* group fed with a commercial diet + 1% of dry nettle, *N3* group fed with a commercial diet + 3% of dry nettle, L* Lightness, a* redness, b* yellowness, *WHC* Water holding capacity

*no statistically significant differences, *P* value > 0.05

### Histomorphology of the duodenum and jejunum

**Fig. 1:**
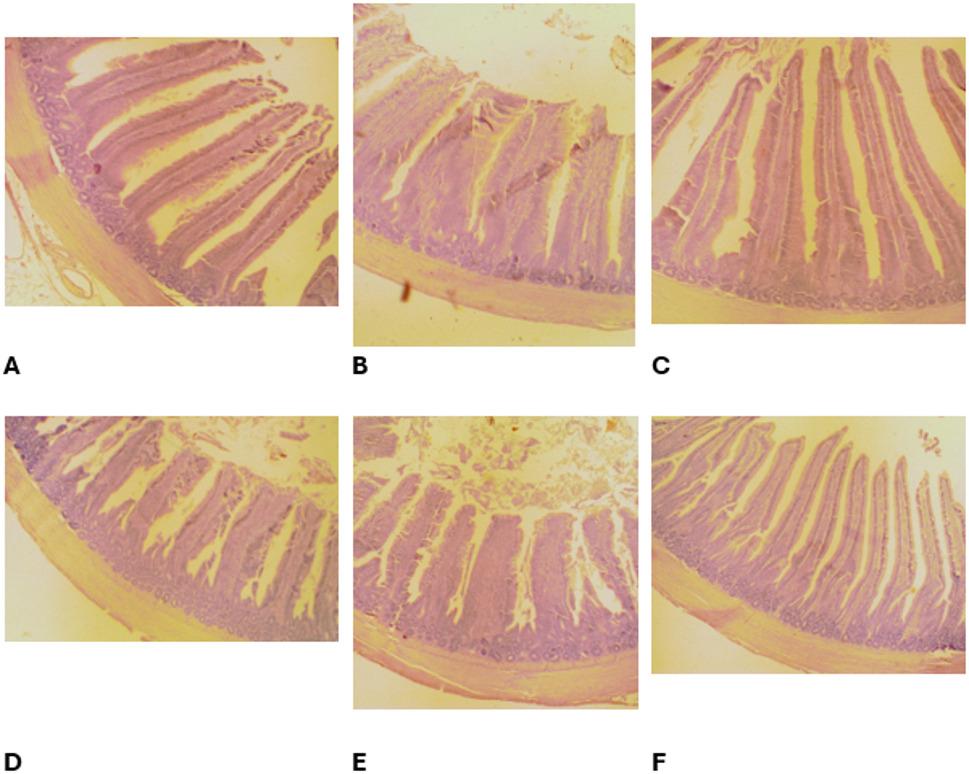
**A**-**F**. Microscopic images of the small intestine of a duck. Duodenum: **A** – group **C**, **B** – group N1, **C** – group N3; Jejunum: **D** – group **C**, **E** – group N1, **F** – group N3. PAS staining, mag. 40x

The highest villus width of the duodenum was recorded in group N3, translating into a significantly larger absorptive surface area than groups 1 and 2 (*P* < 0.05). No statistically significant differences were found between the examined groups regarding villus height and crypt depth. The height of the intestinal villi of the jejunum was significantly greater in groups C and N3 compared to group N1 (*P* < 0.05). In contrast, the greatest villus width was observed in group N1, showing a significant difference compared to group N3 (*P* < 0.05). Parameters such as villus surface area and crypt depth did not differ significantly between the analyzed groups (Table 5; Fig. 1).

**Table 5:** Histomorphology of the duodenum and the jejunum

Item	Group			SEM	*P*-value
	C	N1	N3		
Duodenum					
Villus height (µm)	1028.93	994.84	1095.18	20.014	0.103
Villus width (µm)	175.08^b^	193.19^b^	213.45^a^	4.157	< 0.001
Villus surface area (µm^2^)	572586.10^b^	591867.50^b^	740587.40^a^	19095.600	< 0.001
Crypt depth (µm)	72.23	74.92	78.88	1.617	0.251
Jejunum					
Villus height (µm)	1027.99^a^	934.42^b^	1103.94^a^	16.888	< 0.001
Villus width (µm)	220.48^ab^	242.34^a^	201.93^b^	4.800	< 0.001
Villus surface area (µm^2^)	714174.50	703456.00	704647.50	18139.640	0.762
Crypt depth (µm)	83.34	82.76	85.49	1.617	0.973

*SEM* Standard error of the mean, *C* Control group fed with a commercial diet, *N1* group fed with a commercial diet + 1% of dry nettle, *N3* group fed with a commercial diet + 3% of dry nettle

^a, b^ mean values ​​marked with different letters in the column indicate statistically significant differences between groups, assuming *P* < 0.05

### Viscosity of the digesta of broiler ducks

Analysis of digesta viscosity revealed statistically significant effects related to intestinal segment, dietary nettle supplementation, and their interaction (Table 6). Viscosity was higher in the ileum compared to the jejunum (*P* < 0.001). A significant effect of nettle addition was also observed (*P* = 0.014), with the highest mean viscosity in the control group (3.99 cP) and the lowest in the N3 group (3.65 cP). A significant interaction between nettle inclusion level and intestinal segment was detected. The higher viscosity in the ileum of groups C and N1 compared to the other segments was found, and simultaneously, significantly the lowest viscosity was found in the jejunum from groups N1 and N3 (*P* < 0.001).

**Table 6:** Viscosity of the digesta of broiler ducks

Group			Viscosity (cP)
Nettle effect		C	3.99^a^
		N1	3.82^ab^
		N3	3.65^b^
Intestines		jejunum	3.53^b^
		ileum	4.11^a^
Interaction	C	jejunum	3.82^b^
		ileum	4.17^a^
	N1	jejunum	3.41^c^
		ileum	4.23^a^
	N3	jejunum	3.37^c^
		ileum	3.94^ab^
SEM			0.055
Nettle effect			0.014
Intestine effect			< 0.001
Interaction (nettle × intestine)			< 0.001

*SEM* Standard error of the mean, *C* Control group fed with a commercial diet, *N1* group fed with a commercial diet + 1% of dry nettle, *N3* group fed with a commercial diet + 3% of dry nettle

^a, b,c,^… mean values ​​marked with different letters in the column indicate statistically significant differences between groups, assuming *P* < 0.05

### Relative expression of selected genes in the liver of broiler ducks

**Fig. 2:**
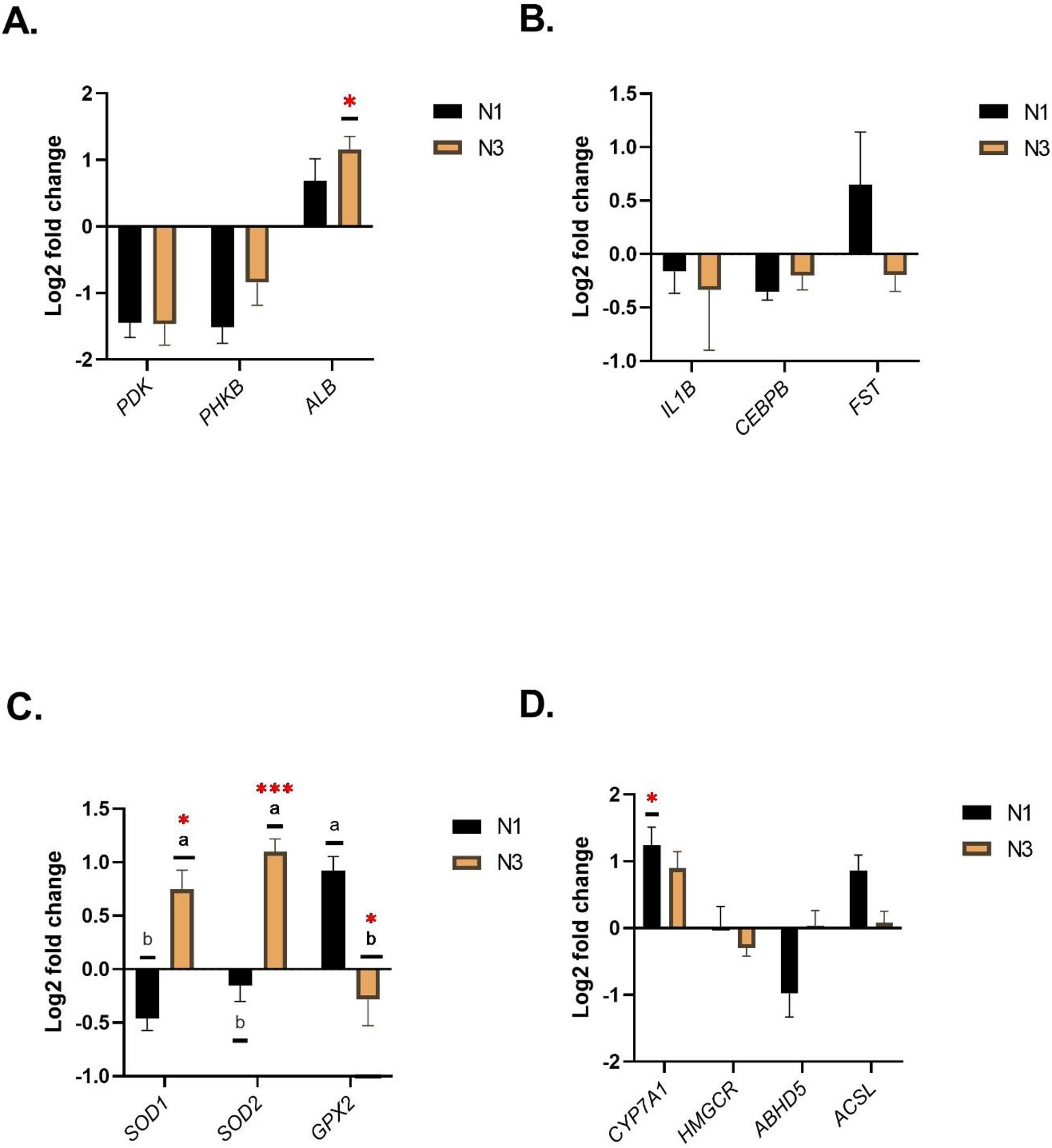
**A**–**D**. The effect of 1% (N1) and 3% (N3) nettle meal supplementation on the expression of selected genes involved in: energy metabolism and carbohydrate regulation (**A**), inflammatory response and cellular metabolism (**B**), neutralization of free radicals and protection against oxidative stress (**C**), as well as lipid and cholesterol metabolism regulation **D** in the liver of broiler ducks Footnotes: N1, group fed with a commercial diet + 1% of dry nettle; N3, group fed with a commercial diet + 3% of dry nettle; statistically significant (*P<0.05) and highly significant (***P<0.01) differences in gene expression between the experimental and control groups are marked with asterisks. Experimental groups labeled with different letters (**a**, **b**) differ significantly. Statistical analyses were performed using one-way ANOVA followed by Tukey’s post hoc test (*P*≤0.05)

The expression of 13 genes associated with oxidative stress, lipid and cholesterol metabolism, inflammation, and carbohydrate metabolism was analyzed in the liver of broiler ducks. The genes were categorized according to their biological functions as follows: *PDK*, *PHKB*, and *ALB* (energy metabolism and carbohydrate utilization; Fig. 2A); *IL-1β* and *CEBPB* (inflammatory response; Fig. 2B); *FST* (cell growth and differentiation; Fig. 2B); *SOD1*, *SOD2*, and *GPX2* (oxidative stress response; Fig. 2C); *CYP7A1*, *HMGCR*, *ABHD5*, and *ACSL* (lipid and cholesterol metabolism; Fig. 2D). Detailed fold change values and statistical comparisons are provided in Table 7.

**Table 7:** Relative gene expression levels of selected genes in experimental groups (N1 and N3) compared to the control group (C)

Gene	N1 (FC)	N3 (FC)	N1 (log2FC)	N3 (log2FC)	C–N1 (*P*-value)	C–N3 (*P*-value)	N1–N3 (*P*-value)
*PDK4*	0.72	0.64	-0.47	-0.64	0.774	0.623	0.966
*PHKB*	0.90	1.32	-0.15	0.39	0.976	0.837	0.718
*ALB*	1.33	2.28	0.41	1.19	0.646	**0.044**	0.234
*IL1B*	0.83	1.71	-0.27	0.77	0.947	0.641	0.453
*CEBPB*	0.84	0.91	-0.26	-0.13	0.460	0.811	0.827
*FST*	1.08	1.46	0.11	0.54	0.969	0.504	0.649
*SOD1*	0.73	1.62	-0.45	0.70	0.141	**0.016**	**< 0.001**
*SOD2*	0.88	2.14	-0.19	1.09	0.701	**< 0.001**	**< 0.001**
*GPX2*	0.76	1.93	-0.40	0.94	0.457	**0.025**	**< 0.002**
*CYP7A1*	2.24	1.81	1.16	0.86	**0.019**	0.096	0.716
*HMGCR*	0.91	1.02	-0.13	0.03	0.932	0.998	0.905
*ACSL1*	2.11	1.33	1.08	0.41	0.070	0.640	0.279

Fold change (FC) values are presented relative to the control group (C) and were calculated using the 2^-ΔΔCt method. Log2FC values correspond to − ΔΔCt. Statistical significance was assessed using ΔCt values with adjusted *P*-values (padj). Significant values (padj < 0.05) are indicated in bold

For antioxidant-related genes, *SOD1*, *SOD2*, and *GPX2* expression did not differ between the control and N1 groups; however, in the N3 group, *SOD1* (*P* = 0.016) and *SOD2* (*P* < 0.001) were significantly higher, whereas *GPX2* was significantly lower (*P* = 0.025; *P* = 0.002) compared to both groups. A significant increase in *CYP7A1* expression, a gene involved in bile acid synthesis and cholesterol catabolism, was detected in the group supplemented with 1% nettle (*P* = 0.019). Although *CYP7A1* expression was also elevated in the 3% nettle group compared to the control, the difference was not statistically significant. No significant differences in *CYP7A1* expression were observed between the two experimental groups.

For *HMGCR*, *ABHD5*, and *ACSL*, no statistically significant changes in expression were found between the control and experimental groups. Although an increase in *ACSL* expression and a decrease in *ABHD5* expression were noted in the N1 group, these changes were not statistically supported and may be attributed to inter-individual variation, as indicated by relatively high standard error values. The expression levels of *IL-1β*, *CEBPB*, and *FST* also did not differ significantly among the experimental groups.

A significant upregulation of *ALB*, a marker of hepatic protein synthesis and metabolic function, was observed in ducks receiving 3% nettle supplementation (*P* = 0.044). In contrast, the expression levels of *PDK* and *PHKB*, which are involved in glucose metabolism and glycogen breakdown, remained unchanged across the analyzed groups.

### The composition of selected bacterial taxa in the cecum of broiler ducks

**Fig. 3:**
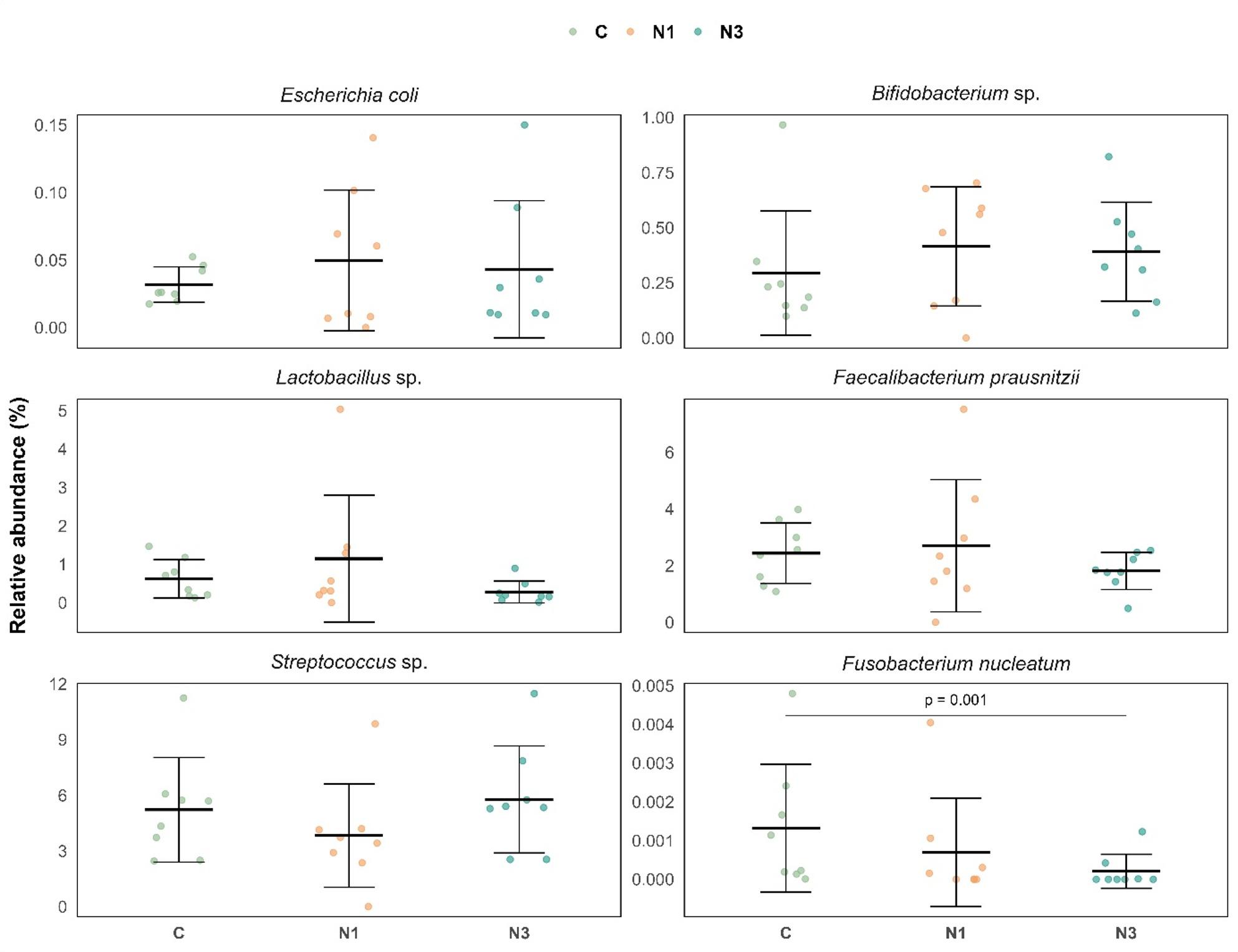
The mean percentage abundance of selected bacterial taxa in the cecal content of broiler ducks Footnotes: C, control group fed with a commercial diet; N1, group fed with a commercial diet + 1% of dry nettle; N3, group fed with a commercial diet + 3% of dry nettle

The Fig. 3 presents the mean percentage abundance of selected bacterial taxa in the cecal content of broiler ducks, depending on the dietary group: control (C) and experimental groups receiving feed supplemented with 1% (N1) or 3% (N3) nettle. The analysis included representatives of the genera *Bifidobacterium*, *Lactobacillus*, *Streptococcus*, and *Salmonella*, as well as the species *Escherichia coli*, *Faecalibacterium prausnitzii*, and *Fusobacterium nucleatum*. 

For most of the analyzed taxa, no statistically significant differences were found between groups. All bacteria were present in low but comparable amounts. The highest relative abundance was observed for *Streptococcus* sp., with the highest levels in group N3 and the lowest in N1. *Lactobacillus* sp. and *Faecalibacterium prausnitzii*, known as beneficial components of the gut microbiota, were most abundant in group N1. In contrast, *Bifidobacterium* sp. and *E. coli* were detected at similar levels across all groups. Neither *Salmonella* sp. nor *Campylobacter jejuni* were detected in any of the samples, indicating high microbiological quality of the rearing environment.

An exception was *Fusobacterium nucleatum*, whose relative abundance decreased significantly with increasing levels of nettle supplementation. The highest abundance of *F. nucleatum* was recorded in the control group (C), lower in N1, and the lowest in group N3. The difference between groups C and N3 was statistically significant (*P* ≤ 0.001), indicating a marked effect of 3% nettle inclusion in the diet in reducing the presence of this species.

## Discussion

In our study, considerable variation in survival rates was observed; however, the differences between groups were not statistically significant (*P* > 0.05). Final body weight and growth performance did not differ significantly among treatments (*P* > 0.05). This contrasts with findings by Mierlita et al. [30], who reported reduced performance in broilers receiving 3% finely ground nettle. Similarly, Biyatmoko et al. [31] observed significant increases in body weight in ducks fed herbal phytobiotic supplements. Feed intake was not significantly affected by nettle inclusion (*P* = 0.058). The highest intake was recorded in group P1 and the lowest in P3. These findings align with earlier studies [32, 33], indicating that herbal phytobiotics do not reduce feed palatability or voluntary intake in poultry. Feed conversion ratio (FCR) also did not differ significantly among groups (*P* = 0.089), though the best FCR was noted in group P1. While Pliego et al. [34] reported improved FCR with 1% nettle inclusion, this effect was not confirmed in the current study.

No significant differences were found in the European Broiler Index (*P* = 0.210) or in carcass yield and its components across groups (*P* > 0.05). Similar findings were reported by Bekele et al. [35], where only selected carcass traits were influenced by nettle leaf inclusion. Physicochemical properties of breast and leg muscles, including pH, color parameters (L*, a*, b*), drip loss, and water-holding capacity, also showed no significant differences between treatments (*P* > 0.05). Minor fluctuations in individual values were observed, but all remained within typical ranges. These results are consistent with Eleroğlu et al. [36], Karre et al. [37], and Marcinčáková et al. [38], who also reported minimal effects of herbal additives on meat quality traits. The lack of significant differences may be attributed to the birds’ high physiological adaptability and the balanced composition of the basal diet, which likely met al.l nutritional requirements regardless of supplementation. Moreover, the doses of nettle used may not have been sufficient to exert strong bioactive effects under the given rearing conditions. These findings suggest that, while nettle is a safe phytogenic additive, its efficacy as a growth performance, carcass, and meat quality enhancer in ducks may be dose-dependent and influenced by diet composition, genetic factors, and environmental stressors.

Histomorphological evaluation of the duodenum and jejunum in broiler ducks supplemented with dry nettle revealed dose-dependent changes in intestinal morphology. In the duodenum, significantly higher villus width was observed in group N3, resulting in an increased villus surface area compared to the 1% nettle group (N1) and the control group (C). Increased intestinal villus width indicates normal mucosal development and may reflect enhanced nutrient absorption [39, 40]. However, in the present study, this did not translate into improved performance parameters.

In the jejunum, a different response pattern to nettle supplementation was noted. Ducks in the C and N3 groups exhibited significantly higher villi than those in group N1, whereas villus width was highest in group N1. This suggests that a lower dose of nettle (1%) may promote villus widening, while a higher dose (3%) is more effective in stimulating villus elongation. Intestinal villi are mucosal projections that undergo structural changes depending on their anatomical location and are essential for increasing the absorptive surface. They are richly vascularized, particularly at their tips, to facilitate nutrient uptake [41]. Villus height is a well-recognized indicator of intestinal health and is often related with improved growth performance and feed efficiency [42, 43]. However, no corresponding improvement in production traits or feed conversion was observed. Sadeeq et al. [14] also reported that non-fermented nettle supplementation at levels of 1.5% and 3% led to increased villus height in the jejunum of broiler chickens, highlighting nettle’s role in mitigating heat stress in poultry.

Interestingly, crypt depth did not differ significantly among the groups in the duodenum or the jejunum. Intestinal crypts are involved in epithelial cell renewal and are sites of defensin production; antimicrobial peptides with immune and endocrine functions [41]. An increase in crypt depth may reflect a regenerative response to inflammation or injury [44]. The absence of significant changes in this parameter suggests that nettle supplementation, even at higher doses, did not induce stress-related or regenerative responses in the intestinal mucosa.

In contrast, villus surface area in the jejunum did not significantly differ among groups, likely due to the inverse relationship between villus height and width observed in this section. This points to a region-specific physiological response to the phytobiotic, with the duodenum appearing more sensitive to dietary nettle. However, these results differ from those of Sadeeq et al. [14], who found that higher nettle inclusion increased the absorptive surface area of jejunal villi. The findings support the potential of phytobiotics as natural growth promoters that enhance intestinal health in poultry production [45]. Nonetheless, the variation in histological responses across different sections of the small intestine suggests that the effects of nettle supplementation are site-specific within the gastrointestinal tract.

A significantly lower intestinal content viscosity was observed in the group receiving the highest level of dried nettle supplementation (3.65 cP) compared to the control group (3.99 cP). Supplementation with dried nettle had a more favorable effect on jejunal content viscosity, in contrast to its effect in the ileum. In the study by Ibrahim et al. [46], dietary inclusion of stevia leaf powder at 10, 20, and 30 g/kg in the feed of Ross broiler chickens significantly reduced intestinal fluid viscosity to 3.16, 3.12, and 3.10 (reported as Pa·s in the original study, likely corresponding to cP values), respectively, compared to the control group (3.56 Pa/second).

Similarly, Tsiouris et al. [47] did not observe significant differences in intestinal viscosity in *Eimeria*-challenged broilers fed a herbal preparation at 1–2 g/kg. Özek et al. [48] observed a reduction in viscosity when using a mixture of essential oils and organic acids. In the present study, the observed decrease in viscosity may be attributed not only to the presence of bioactive compounds but also to alterations in the chemical composition of the diet resulting from nettle supplementation. The inclusion of dried nettle may have influenced the proportions of dietary fiber fractions (e.g., NDF and ADF) as well as the relative content of structural components, which in turn could have modified the physicochemical properties of the intestinal digesta.

Increased viscosity of gastrointestinal contents in poultry is often associated with high levels of non-starch polysaccharides (NSP), primarily arabinoxylans and β-glucans, in the diet [49]. Elevated intestinal viscosity can negatively affect gut morphology, as demonstrated by Yaghobfar and Kalantar [50], who reported shorter intestinal villi in chickens fed wheat- and barley-based diets. However, in the current study, changes in intestinal viscosity did not translate into differences in production performance or body weight gain in ducks. Vidanarachchi et al. [51] noted that increased ileal viscosity may contribute to reduced weight gain in poultry due to adverse changes in jejunal morphology and increased microbial fermentation activity in the intestines. Conversely, our findings suggest that the broiler ducks, particularly those in the control group, did not appear sensitive to elevated intestinal viscosity regarding nutrient absorption efficiency and body weight gain.

The results revealed significant changes in the hepatic expression of genes encoding albumin (*ALB*), superoxide dismutases (*SOD1* and *SOD2*), cytochrome P450 7A1 (*CYP7A1*), and glutathione peroxidase 2 (*GPX2*). Albumin is the main serum protein synthesized in the liver and is widely recognized as a marker of hepatic metabolic function [52]. The observed increase in *ALB* expression may reflect improved liver metabolic activity and overall physiological status, potentially associated with the presence of bioactive compounds in nettle, such as polyphenols [12]. Similar findings were reported by Behboodi et al. [15], who found that supplementation of drinking water with nettle extract (0.25 ml/L) significantly increased serum albumin levels in broilers, suggesting that bioactive compounds such as polyphenols may stimulate hepatic protein synthesis. Although gene expression and protein abundance represent distinct regulatory levels (transcription vs. translation), the consistency between our findings and those of Behboodi et al. [15] may suggest a similar biological response, although direct mechanistic links cannot be confirmed in the present study.

A significant upregulation of *SOD1* and *SOD2* expression in duck livers suggests enhanced first-line antioxidant defense. Superoxide dismutases play a key role in cellular protection against oxidative stress by catalyzing the dismutation of superoxide radicals into hydrogen peroxide [53]. Our findings indicate that nettle supplementation may enhance components of the antioxidant defense system in birds. Ahmadipour and Khajali [11] reported similar effects in broiler chickens, where dietary nettle (1–1.5%) increased hepatic *SOD1* expression. Their study, however, was conducted at high altitude (2100 m a.s.l.), where oxidative stress due to hypoxia is elevated. In contrast, our study was conducted under lowland conditions without environmental stressors, suggesting that the observed effects may occur independently of hypoxia-related oxidative stress. Behboodi et al. [15] also observed increased total serum SOD activity in chickens supplemented with nettle extract, supporting our gene expression data.

In contrast, Sadeeq et al. [14] found no significant changes in SOD activity in the serum of heat-stressed broilers, regardless of nettle form or dose (0, 1.5, or 3 g/kg in feed or water). The lack of effect in their study may reflect differences in administration method or stress conditions, and it’s worth noting that their nettle dose was over ten times lower than ours. The observed increase in the expression of antioxidant defense genes may be associated with the presence of nettle-derived compounds such as flavonoids and vitamin C [12, 54], although their specific contribution cannot be determined without direct compositional analysis of the feed additive.

Surprisingly, *GPX2* expression was reduced. *Glutathione peroxidase 2* (GPX2) is an antioxidant enzyme that detoxifies hydrogen peroxide, protecting cells from oxidative damage [55]. While GPX2 is primarily expressed in the gastrointestinal tract, it is also present in the liver. Its activity is strongly dependent on selenium availability [56]. Together with SOD and catalase, GPX2 forms the first line of antioxidant defense. These enzymes cooperate to prevent and eliminate reactive oxygen species (ROS), either by neutralizing them directly or by decomposing their precursors [57].

This observation, however, requires cautious interpretation, as gene expression alone does not necessarily constitute a direct indicator of oxidative stress intensity. Complementing the analysis with biochemical markers (e.g., lipid peroxidation or antioxidant enzyme activity) would allow for a more comprehensive evaluation of these changes. In this context, the simultaneous upregulation of *SOD1* and *SOD2*, alongside reduced *GPX2* expression, may indicate differential regulation of the antioxidant response. Such a pattern may reflect either altered coordination within the antioxidant system or an adaptive adjustment of its components; however, these possibilities cannot be distinguished based on the present data and require further experimental verification.

Nettle contains multiple antioxidant compounds—including quercetin, phenolic acids, and vitamins C and E—which have been reported to possess ROS-scavenging properties [10, 54, 58, 59]. These compounds may contribute to the overall antioxidant potential of the diet; however, their direct impact on the regulation of specific genes, such as *GPX2*, remains unclear. Although increased SOD activity leads to higher hydrogen peroxide production, its detoxification may involve multiple enzymatic systems, including catalase or other glutathione peroxidase isoforms such as GPX1 [57]. The relative contribution of these pathways, however, was not assessed in the present study.

The lack of direct evidence linking nettle-derived compounds with *GPX2* expression highlights a gap in the literature that warrants further research. While nettle is a well-documented source of antioxidant compounds [54, 60], there is limited data on their impact on *GPX2* gene regulation. Future studies should focus on transcriptional regulation of *GPX2* using in vivo or in vitro models to determine whether nettle bioactives such as flavonoids or phenolic acids modulate its expression and through what mechanisms.

The *CYP7A1* gene, encoding cytochrome P450 7α-hydroxylase, is a key regulator of cholesterol metabolism in the liver. It catalyzes the rate-limiting step in the biosynthesis of bile acids, converting cholesterol into bile acids and enabling cholesterol excretion and lipid homeostasis [61]. Several studies have shown that dietary bioactive compounds can modulate *CYP7A1* expression. For example, Zhang et al. [62] demonstrated that quercetin upregulated *CYP7A1* in Wistar rats via activation of the LXRα signaling pathway, enhancing bile acid synthesis and cholesterol elimination. Similar effects were observed with chlorogenic acid, which increased *CYP7A1* expression in 129/Sv mice with ANIT-induced cholestasis [63]. The consistency of these findings with our results may suggest a comparable mode of action, although this cannot be directly confirmed in the present experimental model.

As a rich source of polyphenols, including quercetin and chlorogenic acid, nettle may contribute to the modulation of cholesterol metabolism through diet-related mechanisms [62, 63]. Our observation of increased *CYP7A1* expression in nettle-supplemented ducks is consistent with the involvement of bile acid biosynthesis pathways [61]. Nettle supplementation may therefore support cholesterol metabolism, which is important not only for bird health but also for poultry production efficiency. Safamehr et al. [10] reported that nettle-supplemented diets reduced serum cholesterol and triglyceride levels in Ross 308 broilers, while Mansoub [9] found similar effects in laying hens, with 2% nettle supplementation lowering cholesterol by 5.9% and triglycerides by 13.3% compared to controls. These effects may be associated with changes in CYP7A1 expression; however, direct causal relationships require further validation.

Reducing lipid content in poultry tissues through dietary polyphenols may also improve meat quality by decreasing fat content and improving its lipid profile—an important benefit for consumer health. Therefore, our results, along with the findings of Safamehr et al. [10] and Mansoub [9], suggest that nettle supplementation is a promising dietary strategy to reduce lipid accumulation in poultry.

This study evaluated the effect of dietary supplementation with common nettle on the expression of selected genes related to the health status of broiler ducks. The results revealed significant changes in hepatic expression of *ALB*, *SOD1*, *SOD2*, *CYP7A1*, and *GPX2*. Albumin is the main serum protein synthesized in the liver and a marker of hepatic metabolic function [52]. The observed increase in *ALB* expression may reflect improved liver metabolic activity, potentially associated with bioactive compounds present in nettle, such as polyphenols [12]. Similar results were reported by Behboodi et al. [15], who observed increased serum albumin in broilers supplemented with nettle extract. Although gene expression does not directly reflect protein levels, these findings may suggest a comparable biological response.

Upregulation of *SOD1* and *SOD2* suggests enhanced first-line antioxidant defense. Superoxide dismutases catalyze the conversion of superoxide radicals into hydrogen peroxide [53]. Our findings indicate that nettle supplementation may enhance antioxidant defense mechanisms. Similar effects were observed by Ahmadipour and Khajali [11], although under hypoxic conditions. In contrast, our study was conducted under standard conditions, suggesting that these effects may occur independently of environmental stress. Supporting this, Behboodi et al. [15] also reported increased serum SOD activity. Sadeeq et al. [14] found no changes in *SOD* activity in heat-stressed broilers, possibly due to lower nettle dosage or differences in experimental conditions. The observed gene expression changes may be associated with bioactive compounds such as flavonoids and vitamin C [12, 54], although their specific contribution cannot be confirmed without compositional analysis. In contrast, *GPX2* expression was reduced. GPX2 detoxifies hydrogen peroxide and is part of the first line of antioxidant defense [55–57]. This finding should be interpreted cautiously, as gene expression alone is not a direct indicator of oxidative stress. Additional biochemical markers (e.g., lipid peroxidation or enzyme activity) would be required for comprehensive evaluation.

The simultaneous upregulation of *SOD1***/***SOD2* and downregulation of *GPX2* may indicate differential regulation of antioxidant pathways. This pattern may reflect altered coordination or adaptive adjustment within the antioxidant system; however, these possibilities cannot be distinguished based on the present data. Nettle contains antioxidant compounds, including polyphenols and vitamins C and E, with known ROS-scavenging properties [10, 54, 58–60]. These may contribute to the overall antioxidant potential of the diet, although their direct role in regulating genes such as *GPX2* remains unclear. Detoxification of hydrogen peroxide may involve alternative systems, including catalase or other glutathione peroxidases such as GPX1 [61]. The lack of direct evidence linking nettle components with *GPX2* regulation highlights a gap in the literature. Future studies should investigate these mechanisms using targeted experimental approaches.

The *CYP7A1* gene plays a key role in cholesterol metabolism by regulating bile acid synthesis [66]. Its increased expression in our study is consistent with previous findings showing that polyphenols such as quercetin and chlorogenic acid can upregulate *CYP7A1* [62, 63]. This may suggest a diet-related modulation of cholesterol metabolism. Consistent with this, previous studies reported reduced serum cholesterol and triglycerides following nettle supplementation [9, 10]. These effects may be associated with changes in *CYP7A1* expression, although causal relationships require further validation.

In the present study, dietary supplementation with nettle had no significant effect on the relative abundance of bacteria belonging to the genera *Lactobacillus*, *Streptococcus*, and *Bifidobacterium*, nor on the species *Escherichia coli* and *Faecalibacterium prausnitzii* in the cecal content of ducks. This finding suggests a lack of a pronounced modulatory effect of nettle on the analyzed bacterial taxa. At the same time, it should be emphasized that the qPCR-based approach applied in this study provided information only on selected bacterial taxa and did not reflect the overall structure or diversity of the microbiome. Similarly, no effect of nettle extract on the probiotic bacterium *Lactobacillus plantarum* was reported by Kukrić et al. [64], which may indicate that certain commensal bacteria are less susceptible to plant-derived compounds or remain protected within the complex intestinal environment. Alternatively, this may reflect a selective rather than broad-spectrum mode of action of the bioactive compounds present in nettle.

Despite the lack of effect on bacteria generally considered beneficial, supplementation with 3% nettle significantly reduced the abundance of *Fusobacterium nucleatum*, a potentially pathogenic bacterium associated with periodontal disease, ulcerative colitis, and colorectal cancer [65–68]. A similar trend was observed in the study by Zhou et al. [69], where the application of a formic acid polymer in broilers challenged with LPS resulted in a reduction of bacteria from the genera *Fusobacterium*, *Helicobacter*, and *Enterococcus*. Therefore, the observed decrease in *Fusobacterium nucleatum* may be interpreted as evidence of selective modulation of specific taxa rather than a broad-spectrum antimicrobial effect. It is noteworthy that nettle contains natural organic acids, including formic acid, which may acidify the intestinal environment, support digestive processes, and inhibit the growth of pathogenic bacteria [54, 60, 70]. The present findings are partially consistent with reports describing the antimicrobial activity of various *Urtica dioica* preparations, such as leaf powder, extracts, and essential oils, tested in vitro against both Gram-positive and Gram-negative bacteria [65, 71–75]. However, it should be noted that these studies were predominantly conducted on laboratory strains under strictly controlled in vitro conditions, whereas the present study was performed in vivo. Under physiological conditions, the composition of the gut microbiota is shaped by complex interactions among microorganisms, host physiology, and environmental and dietary factors, which may substantially modify or limit the direct effects of plant-derived compounds.

Importantly, evidence from in vivo studies remains inconsistent. For instance, supplementation with nettle seeds (5–15 g/kg feed) in broilers was associated with a reduction in aerobic bacteria and coliforms, although the effect on *Lactobacillus* spp. was ambiguous due to high variability in the data [76]. In another study, dietary inclusion of nettle (9%) in a high-fat diet in mice increased the abundance of *Clostridium* species (e.g., *C. vincentii*, *C. disporicum*) and *Turicibacter*, which was linked to improved metabolic parameters but did not restore the reduced levels of bifidobacteria [77]. Furthermore, a literature review by Harrison et al. [78] highlighted that many studies investigating the antimicrobial activity of nettle are affected by methodological limitations, including the lack of appropriate controls, insufficient information on solvents used, and variability in extraction procedures. Moreover, only a subset of these studies provided reliable minimum inhibitory concentration (MIC) data, and the authors’ own experiments did not demonstrate antimicrobial activity of nettle leaf extracts against *Staphylococcus aureus* and *Pseudomonas aeruginosa*. This suggests that the antimicrobial effects of nettle are highly context-dependent and not consistently reproducible.

In summary, dietary supplementation with 3% dried *Urtica dioica* positively influenced selected physiological and metabolic parameters in broiler ducks without adversely affecting growth performance, carcass yield, or meat quality. Although production traits remained unchanged, improvements were observed in duodenal and jejunal histomorphology and digesta viscosity. At the molecular level, nettle upregulated genes associated with antioxidant defense, hepatic protein synthesis, and cholesterol metabolism, indicating enhanced liver function and oxidative status. It also preserved beneficial cecal microbiota while reducing *Fusobacterium nucleatum*, suggesting a selective antimicrobial effect. Overall, these findings support the use of dried nettle as a safe phytogenic feed additive that may enhance gut health and physiological resilience, although digestive tract changes were not reflected in production outcomes.

## Data Availability

All datasets supporting the conclusions of this article are included within the article.

## Abbreviations

C: Control group fed with a commercial diet
N1: Roup fed with a commercial diet + 1% of dry nettle
N3: Group fed with a commercial diet + 3% of dry nettle
DM: Dry matter
ADF: Acid detergent fiber
NDF: Neutral detergent fiber
ADL: Acid detergent lignin
BW: Body weight
BWG: Body weight gain
FI: Feed intake
FCR: Feed conversation ratio
L*: Lightness
a*: Redness
b*: Yellowness
WHC: Water holding capacity
SEM: Standard error of the mean
